# Do randomized clinical trials with inadequate blinding report enhanced placebo effects for intervention groups and nocebo effects for placebo groups? A protocol for a meta-epidemiological study of PDE-5 inhibitors

**DOI:** 10.1186/2046-4053-1-54

**Published:** 2012-11-14

**Authors:** Frederik Feys, Geertruida E Bekkering, Kavita Singh, Dirk Devroey

**Affiliations:** 1 Huisartsgeneeskunde, Vrije Universiteit Brussel, Brussel, Belgium; 2 CEBAM Belgian Center of Evidence-based Medicine vzw, Leuven, Belgium; 3 The Centre for Practice-Changing Research, Ottawa Hospital Research Institute, Ottawa, Canada

**Keywords:** Bias, Blinding, Expectancy, Nocebo, Phosphodiesterase-5 inhibitor, Placebo, Randomized controlled trial

## Abstract

**Background:**

Patients’ expectations of treatment effects may contribute to positive (placebo) and negative (nocebo) outcomes. The effect of patient expectations may be pronounced in subjectively assessed conditions, such as male erectile dysfunction. The aim of this project is to examine the magnitude of expectancy in trials of phosphodiesterase-5 inhibitors. We hypothesize that randomized controlled trials with inadequate blinding will report enhanced placebo effects for intervention groups and nocebo effects for placebo groups, compared with adequately blinded studies.

**Methods/design:**

We will quantify the magnitude of expectancy by comparing the effect estimates of trials with inadequate and adequate blinding. Blinding will be assessed using four domains from the Cochrane ‘risk-of-bias’ tool: allocation concealment; blinding of patient; caregiver; and outcome assessor. Our secondary aim is to identify factors that can modify expectations, such as prior experience with the intervention and drug side effects.

We will perform an electronic search using a combination of controlled vocabulary and free text words in the following databases: MEDLINE, EMBASE, CENTRAL, and a clinical trials register. We will include randomized controlled trials, with either parallel or crossover design, that compare one phosphodiesterase-5 inhibitor with a placebo. The study’s primary aim should be to investigate the efficacy of phosphodiesterase-5 inhibitors for treating male erectile dysfunction. Screening will take place at two levels: abstracts and titles, followed by full text reports. Two reviewers will independently extract data on the primary outcome and assess risk of bias.

We will meta-analyze treatment effects, if appropriate, to assess the magnitude of enhanced placebo effects and nocebo effects in intervention and placebo groups, respectively. We will explore possible mediators of placebo and nocebo effects with subgroup and meta-regression analyses.

**Discussion:**

Treatments may confer significant costs and risk of adverse effects; it is important, therefore, to determine whether the effects of treatments are larger than expectancy alone. If treatment expectations can be used in a non-deceptive way to produce clinically advantageous outcomes, then it may be possible to incorporate such mechanisms into evidence-based healthcare decision-making.

## Background

A particular treatment effect may exert both non-specific and specific effects. A non-specific treatment effect is an outcome that does not arise according to an intended mechanism of action. This can be a response to a placebo but can also reflect a spontaneous symptom improvement. A placebo is usually thought of as a sugar pill, but placebos can come in any form; they may be things (syringes, medical devices), rituals (anamnesis, ingestion of drugs), places (hospital, doctor’s office), relationships (with doctor, self-help group), medical beliefs to suggestive wordings [1]. The response to a placebo can be either positive for the outcome of interest, defined as a placebo effect, or negative for the outcome of interest, defined as a nocebo effect.

These effects are commonly explained by expectancy and conditional learning [2]. These two concepts overlap, so for convenience, we will use the term ‘expectancy’ to describe the mechanism behind placebo and nocebo effects.

Studies have shown that expectations can induce very powerful effects. In an experiment with an opioid painkiller, remifentanil, the presence of positive treatment expectancies was found to double the analgesic effect. Conversely, negative treatment expectancies interfered with the analgesic potential of the painkiller to the extent that the analgesic effect was completely abolished [3]. In a double-blind sham surgery trial, investigating a new surgical transplant technique for treatment of Parkinson’s disease, sham and real surgery interventions were equally effective. However, participants who thought they received the transplant reported better quality of life [4]. It seems that positive expectations were triggered by the perceived benefit from the treatment.

To control for the effects of expectations, the double-blind randomized controlled trial (RCT) design is commonly employed to study a novel intervention for its specific effects. Because neither participants nor investigators know who gets the intervention or the placebo, expectancies are balanced across groups. Double blinding makes groups comparable so that specific and non-specific treatment effects (that is, the effect size of the placebo group) can be ascertained with less potential for bias. Both intervention and placebo groups may have two important expectations in common: ‘I get the intervention or the placebo,’ and ‘The intervention under study can cure my problem.’

However, there is little evidence that RCTs are, in fact, double-blinded [5]. Many factors can undermine double-blinded methodology, including poor randomization methods, imperfect concealment of allocation, and the use of a placebo that is distinguishable from the intervention. Furthermore, in RCTs of pharmacological agents, the presence of side effects may allow participants or investigators to guess correctly who has been allocated to intervention or placebo [6]. Therefore, the use of an active placebo that mimics some of the intervention’s side effects has been advocated to improve clinical trial blinding.

If an RCT is not double-blinded, participants and investigators will know who gets what type of treatment. Expectations, therefore, could become unbalanced among treatment arms. A participant allocated to the intervention would have altered expectations: ‘I get the intervention’ and ‘The intervention under study can cure my problem.’ This enhances the participant’s prior expectations and can generate an enhanced placebo effect. For participants receiving the placebo, expectations could be ‘I get the placebo,’ and ‘The intervention under study can cure my problem.’ This can lower participants’ expectations and generate a nocebo effect.

This review will test the hypothesis that unblinding in RCTs is associated with enhanced placebo effects for intervention groups and nocebo effects for placebo groups. We will investigate this research question by conducting a meta-epidemiological study of phosphodiesterase-5 (PDE-5) inhibitors. For many years, this treatment has been an established baseline treatment for erectile dysfunction (ED). Numerous trials, overviews, and systematic reviews provide evidence for the efficacy and safety of sildenafil, tadalafil, and vardenafil. There is also a growing evidence base for the newer molecules mirodenafil, udenafil, lodenafil, and avanafil. The PDE-5 inhibitors have been tested in many different populations, including those with broad-spectrum and specific comorbid conditions. The role of treatment expectations is of particular relevance to these medications for several reasons. Firstly, the evidence for efficacy relies solely on subjectively assessed outcomes, such as self-administered questionnaires (International Index of Erectile Functioning (IIEF)), event logs, and a global efficiency question (GEQ) [7]. Randomized control trials that use these subjective outcome measures are especially vulnerable to unblinding: non-blinded RCTs report 25% higher estimates of treatment effects than their blinded counterparts [8]. Whether this can be explained by nocebo effects in placebo groups or enhanced placebo effects for intervention groups was not reported. Secondly, since PDE-5 inhibitors are a well-tolerated and effective treatment for ED, initial expectations to treat this common male sexual problem are high for doctors, patients, and drug companies. Lastly, as suggestion can create expectancies [9], the domain of male sexual performance is a very suggestive domain, where expectations can play a fundamental role.

This meta-epidemiological study will explore magnitude of expectancy and mediating factors in RCTs. If the mechanisms mediating placebo effects such as expectations can be used in a non-deceptive way to produce clinically advantageous outcomes, then it may be possible to incorporate these mechanisms into evidence-based healthcare decision-making.

## Methods/design

### Literature search

We will use data from an earlier published systematic review on PDE-5 inhibitors that included RCTs up to July 2006 [10]. This dataset will be updated for any missing variables and for new reports. To ease clinical interpretation of our findings, we will restrict our search to the three agents approved by the Food and Drug Administration (FDA): sildenafil, vardenafil, and tadalafil. We will use the following methods to identify studies reported in English, French, Dutch, or German since July 2006.

Our search strategy will employ a combination of controlled vocabulary (MeSH terms) and free text words. Three concepts will be combined (AND): the intervention concept ‘PDE-5 inhibitor’, the disease concept ‘erectile dysfunction,’ and the design concept ‘RCT.’ The latter will be identified using the Cochrane Highly Sensitive Search Strategy for identifying RCTs [11]. We will search MEDLINE using PubMed, EMBASE, CENTRAL (The Cochrane Central Register of Controlled Trials) and a clinical trials register (clinicaltrials.gov). The PubMed/MEDLINE search strategy is presented in Appendix 1 and will be adapted to search the other electronic databases.

### Selection of studies

One reviewer (FF) will manually screen the titles and abstracts of search yields using pre-defined screening questions to remove studies that are obviously irrelevant to our topic (Appendix 2). The full texts of potentially relevant reports will then be obtained. If multiple reports for the same study exist, they will be linked to determine eligibility. Two reviewers, a content area expert (FF) and a methodologist (GEB), will independently screen full, unblinded texts using a pilot-tested eligibility form (Appendix 3). If there is disagreement among reviewers, it will be recorded and resolved by discussion. The level of agreement among reviewers will be quantified with a kappa statistic. We will describe the selection of studies in a flow diagram, as recommended by the PRISMA statement (preferred reporting items for systematic reviews) [12], and keep a list of excluded studies, with primary reasons for exclusion.

We will screen studies according to the following inclusion criteria:

•Study design: RCTs with parallel or crossover design that compare one PDE-5 inhibitor to placebo. The study’s primary aim should be to investigate the efficacy of PDE-5 inhibitors in treating male ED.

•Participants: Men ≥ 18 years old complaining of or diagnosed with ED.

•Intervention: Treatment for ED with a PDE-5 inhibitor (sildenafil, vardenafil, tadalafil) at any dose regimen.

•Comparison: placebo.

•Study reports numerical values for change from baseline or baseline and final IIEF erectile functioning (EF) scores for the placebo and intervention groups separately.

### Data extraction and risk-of-bias assessment

We will develop and pilot test electronic forms to extract data and assess risk of bias of all included study reports. Items to be included in the data extraction form are provided in Appendix 4. We will assess blinding using four domains of the Cochrane’s ‘risk-of-bias’ tool that relate to blinding: allocation concealment, blinding of patient, blinding of caregiver, and blinding of outcome assessor. In addition, the risk-of-bias assessment will include an evaluation of sequence generation, intention-to-treat (ITT) analysis (quantified as the ratio of analyzed versus allocated number of participants) and comparability of treatment and placebo groups (for example, assessment of baseline prognostic characteristics). For each included study, we will rate the risk-of-bias domains as low, high, or unclear. To improve accuracy, all primary outcome data and risk-of-bias assessments will be independently extracted by a second reviewer (GEB). Disagreements will be recorded and resolved by open discussion.

### Data analysis

#### Quantifying enhanced placebo and nocebo effects

We will quantify the magnitude of enhanced placebo effects as the difference in treatment effect estimates among studies with inadequate blinding and studies with adequate blinding. Similarly, the magnitude of nocebo effects will be quantified as the difference in placebo effect estimates among studies with inadequate and adequate blinding.

#### Outcomes

The most commonly used outcome in efficacy studies of PDE-5 inhibitors is the IIEF-EF domain score [13]. Therefore, we will use this outcome to assess effect sizes. We will also assess GEQ, a common dichotomous outcome, to confirm consistency of the effect. Our primary and secondary outcomes are:

1. Primary: the IIEF-EF change from baseline score from the validated IIEF questionnaire for placebo and intervention groups separately (a continuous subjective outcome assessed by patient). For the treatment effect size, the mean difference (MD) with 95% confidence intervals (CI) between change from baseline IIEF-EF-score_Intervention group_ and the change from baseline IIEF-EF-score_Placebo group_ will be calculated. If only final and baseline IIEF-EF scores are reported, we will assume change scores to be final-minus-baseline scores. A sensitivity analysis for this assumption will be performed.

2. Secondary: for placebo and intervention groups separately:

2.a. GEQ (a dichotomous subjective outcome assessed by patient). The risk ratio (RR) with 95% CIs will be calculated.

2.b. Type and number of adverse events (AEs) reported. At least the two most common AEs reported for PDE-5 inhibitors: headache and flushing (a continuous subjective outcome assessed by clinician). The RR with 95% CIs will be calculated. Only AE incidences, measured as single events (that is, no count data), will be extracted.

For GEQ, all randomized participants will be included in the analysis, irrespective of how the authors of the report defined their ITT sample; therefore, all discontinuations from the point of randomization will be considered non-response.

We will impute missing data, such as standard deviation, based on other available data, such as standard error, 95% CI, *t* value, or *P* value. If imputation of missing data is not possible, we will contact the original investigators to request missing data. If there is no response, we will use data from matched studies.

Crossover study designs that report only first-phase outcome data will be treated as a parallel RCT and included in the main analysis. Studies that report only final-outcome data will be included in a separate crossover study analysis. For studies that report data for two phases separately, we will calculate the difference between first and second phases and use a *t* test for statistical confirmation. If we find no difference, then we will pool data and include them in a separate crossover studies analysis. If we find a difference, then we will extract only first-phase data and treat the study as a parallel study design to be included in the analysis.

As a secondary objective, we will explore moderating variables of placebo and treatment effect estimates that may explain enhanced placebo and nocebo effects. Of particular interest are prior experience with medication, drug side effects, exclusion of placebo responders, study run-in period, sample size, geographical location of the study, single- or multi-center study, risk of bias, proportion of psychogenic etiology, prostate cancer or spinal cord injury, funding source, publication year, baseline disease severity, disease duration, study duration, and number of follow-ups after baseline assessment.

#### Data synthesis

We will meta-analyze studies, if appropriate, using generic inverse variance. We plan to use a random-effects model because we anticipate that the included studies will show considerable clinical (broad-spectrum and specific comorbid populations; different PDE-5 inhibitors) and methodological (study design, risk of bias) heterogeneity. The analysis will include all parallel RCTs and a separate analysis will include crossover RCTs. For the latter, we anticipate that carry-over effects can contribute to unblinding. Therefore, lower placebo and higher treatment effects in crossover studies may be present than in parallel studies only. We will pool data from both study designs if no significant differences are found between the separate analyses.

Variability in effect estimates that are due to heterogeneity rather than sampling error (that is, chance) will be identified visually using a forest plot. The magnitude of heterogeneity will be assessed by calculating *I*^2^ with confidence interval and confirmed statistically with a chi-square test with 0.10 significance level.

For every individual risk-of-bias domain, we will group studies with low risk of bias and studies with unclear or high risk of bias. Studies that have a low risk of bias across all four risk-of-bias domains, and therefore considered adequately blinded, will be pooled and compared with studies that have a high or unclear risk of bias across all four risk-of-bias domains (Table 1). For both groups, we will calculate pooled treatment and placebo effect. The difference in effect estimates of placebo groups between the two sets of studies will be the nocebo effect and the difference in treatment effect estimates of intervention groups will be the enhanced placebo effect. Differences between groups will be quantified with a 95% CI and then qualified statistically using a *t* test of no difference with *P* value. Between-meta-analysis heterogeneity variance will be calculated to express the variability in bias with *P* value and identified visually using a forest plot. The magnitude of heterogeneity will be assessed by calculating *I*^2^ and confirmed statistically with a chi-square test with 0.10 significance level. We will assess reporting bias by visually examining a funnel plot for symmetry if there are a sufficient number of studies.

**Table 1:** Enhanced placebo effect and nocebo effect

**IIEF-EF change scores**		**Placebo effects**				**Treatment effects**				
**Risk-of-bias domain**	Number of trials	Studies with low risk of bias	Studies with unclear or high risk of bias	Difference^(A)^	*P* value (*t* test)	Studies with low risk of bias	Studies with unclear or high risk of bias	Difference^(B)^	*P* value (*t* test)	Variability in bias (*P* value)
**Allocation concealment**	*n*/*N*	*a* (95% CI) (IV)	*b* (95% CI) (IV)	*b* − *a* (95% CI)		*c* (95% CI) (IV)	*d* (95% CI) (IV)	*d* − *c* (95% CI)		
**Blinding: participant**	…	…	…	…		…	…	…		
**Blinding: caregiver**	…	…	…	…		…	…	…		
**Blinding: outcome assessor**	…	…	…	…		…	…	…		
**Summary of four risk of bias domains**	…	…	…	…		…	…	…		

CI, confidence interval; IV, inverse variance.

The comparison of adequately and inadequately blinded studies is observational and, therefore, blinding status is likely to be associated with other variables that also influence within group treatment and placebo effect estimates (that is, the association between blinding status and effect estimates will be confounded). Using meta-regression analysis, we will investigate baseline ED severity and publication year as possible confounders and present adjusted effect estimates for enhanced placebo effect and nocebo effect.

To interpret nocebo effects in placebo groups and enhanced placebo effects in intervention groups, we will conduct the following analyses: examine forest plots stratified according to risk of bias; interpret *P* value on lack of these effects (*P* < 0.05 significance) and variability in bias between subgroups; and explore clinical relevance of these effects using reported minimal clinically important differences (MCID) for IIEF-EF scores [14].

#### Assessment of adverse events on nocebo and enhanced placebo effects

We expect a limited number of studies to be adequately blinded so, for power considerations, we will explore solely whether AEs can explain nocebo and enhanced placebo effects. The type of AE that placebo groups report seem to match AEs of intervention groups [15]. This is sometimes explained as a nocebo effect due to the informed consent document. This document informs study participants of the most common AEs (in the case of PDE-5 inhibitors, the most common AEs are headache and flushing) and sets out AE expectations that can modify the rates of reported AEs [16]. This reporting effect may also be enhanced by AE expectations of outcome assessors. Differing AE rates in placebo and intervention groups are believed to be a major determinant in unblinding RCTs. Study participants reporting many AEs may also enhance their expectations of receiving the intervention under study. This could explain enhanced placebo effects. Conversely, lack of AEs can lower expectations of receiving the intervention under study, creating a nocebo effect. More concordant AE rates (mathematically, we will use RRs) for placebo and intervention groups may render the study less prone to unblinding. Groups of studies with higher risk of unblinding may be associated with higher RRs and thus higher nocebo effects in placebo groups and enhanced placebo effects in intervention groups.

#### Meta-regression and subgroup analyses

We will use meta-regression univariate and multivariate analysis to investigate patient- and study-related variables that influence placebo and treatment effects. The following variables will be explored:

1. Number of study follow-ups after baseline assessments. Every follow-up involves an interaction between study personnel and the participant. Any (un)conscious modification of treatment outcome expectation can potentially invalidate the double-blind procedure. More frequent contact would then translate into enhanced placebo effects, seen as higher treatment effects, and nocebo effects, seen as lower placebo effects [17].

2. Sample size.

3. Study duration.

4. Proportion of psychogenic etiology. Some evidence suggests that participants having ED due to psychological mechanisms report higher placebo effects.

5. Prostate cancer or spinal cord injury. These two conditions are associated with low expectancy of EF. We expect lower placebo and treatment effects.

6. Baseline ED severity (IIEF-EF score)

7. ED duration. The longer the ED exists, the lower expectancy of recovery.

8. ITT analysis. Studies excluding participants from analyses can result in biased effect sizes.

9. Publication year. Early reports can reflect data from studies that have higher expectations about the new treatment.

We will carry out subgroup analysis to measure the impact of study, intervention, and patient factors on placebo effects and treatment effects separately. To investigate whether the effect is different in the case of subgroups, the overlap in CIs of summary estimates will be considered. An *I*^2^ statistic for between-subgroup heterogeneity will be calculated and a significance test performed. If a sufficient number of studies can be included in our main analysis, we may conduct some of the following subgroup analyses, with priority for the factor ‘prior PDE-5 inhibitor experience’.

Study factors:

1. Parallel studies only.

2. Crossover studies only. We anticipate that carry-over effects can contribute to unblinding during RCTs. Therefore, lower placebo and higher treatment effects in crossover studies may be present than for parallel studies only.

3. Study run-in phase with placebo (yes/no). During a study run-in phase with placebo, participants and study personnel know that placebo is given for every participant. When the double-blind phase starts, some participants are switched over to the intervention and the remaining participants stay on placebo. It can be hypothesized that the former group suddenly experiences change of bodily cues, owing to pharmacological effects of the intervention (such as AEs), potentially augmenting their belief that they are receiving the intervention. It can be expected that this group report a larger treatment effect, owing to higher expectations of treatment benefit in comparison with an intervention group that had no placebo run-in phase. In placebo groups, prior experience with placebo is expected to lower effects.

4. Commercial funding (yes/no). Commercially funded studies may have a lower incentive to protect study quality because higher study quality is associated with smaller treatment effects [18]. We expect commercial funding to be associated with lower placebo and higher treatment effects.

5. Continent (America, Europe, Australia, Asia). Some evidence suggests that placebo effects differ according to geographical locations [19].

6. Single-center study (yes/no). In multi-center studies, a centrally regulated organization conducts important aspects of RCTs, such as generation of randomization sequences, concealment of allocations, and the preparation of study drugs. In single-center studies, participants and study personnel all meet in one location, which increases opportunities for information exchange and consequent interference with double-blinded procedures. We expect single-center studies to be associated with lower placebo and higher treatment effects.

Intervention factors:

1. Type of PDE-5 inhibitor: expectations may be higher for sildenafil since this was the first PDE-5 inhibitor on the market.

Patient factors:

1. Prior PDE-5 inhibitor experience. Prior exposure to effective pharmacological agents has been found to produce very strong placebo responses in different pathological conditions, such as Parkinson’s disease, immune response, hormonal secretion, and respiratory depression [20]. We expect prior PDE-5 inhibitor experience to be associated with lower placebo effects and higher treatment effects.

In the case of considerable heterogeneity between study results that cannot be explained by *a priori* defined subgroup and meta-regression analyses, a series of *a posteriori* meta-regression analyses will be performed to identify sources of heterogeneity. *A priori* and *a posteriori* analyses will be labeled as such.

#### Sensitivity analyses

We will perform a sensitivity analysis for different assumptions about missing data. We did not pre-specify the random sequence generation risk-of-bias domain as part of our formal definition of adequately blinded trials. Therefore, we will conduct a sensitivity analysis by including this characteristic in a meta-regression analysis. Lastly, we will compare the results of fixed and random-effects analyses.

## Discussion

This meta-epidemiological study will test the hypothesis that unblinding in RCTs can raise expectations in intervention groups and lower expectations in placebo groups. Additional subgroup analyses and meta-regression will explore factors that can mediate expectancies in RCTs with special emphasis on the role of AEs. This information can be used to focus future methodological and clinical research to better understand the nature and magnitude of expectancy in health outcomes. Given the high cost of many treatments and the cumulative risks of AEs, it is crucial to address whether treatments are substantially more effective than expectancy alone. Methodological rigor in the conduct and reporting of RCTs is especially imperative for those health conditions that are evaluated subjectively, such as depression, ED, low back pain, and other symptom driven complaints.

### Strengths and limitations

To our knowledge, this will be the first study to investigate enhanced placebo and nocebo effects in RCTs. We will examine the impact of risk of bias due to unblinding on a subjective continuous outcome for male ED and we will seek confirmation of findings for an additional dichotomous outcome. Furthermore, this review will be the first to explore the role of AEs in unblinding RCTs. We will include crossover studies to improve the generalizability of our findings. Our methods will be rigorous, including use of pre-defined screening forms, pilot-tested data extraction and risk-of-bias forms, trained reviewers with clinical or methodological expertize, and *a priori* statistical analyses. We will prepare the review according to the PRISMA statement [12].

One possible limitation of the review is that we will use reported study data only, which may not be an accurate representation of actual study conduct: reporting can be poor but study conduct can be good, and vice versa. Secondly, we are evaluating only one class of intervention in the field of male sexual dysfunction, albeit across a comprehensive set of studies. Lastly, we will not search grey literature sources, which may result in some relevant studies being missed. However, an assessment of publication bias will provide an indication of whether unpublished studies are absent from our evidence base.

### Implications of the research

Clinical trials attempt to ascertain the specific isolated effects of interventions. However, there is little quantitative evidence available about the impact of non-specific effects on outcomes. Consequently, these effects are generally ignored in clinical decisions. However, accumulating research demonstrates that non-specific effects are real and significant, especially in subjectively assessed medical conditions. The findings of this study will provide insight into the magnitude of non-specific treatment effects in male ED. By quantifying nocebo and enhanced placebo effect estimates, we hope that clinicians will be able to incorporate such effects in treatment decisions and patient counseling information. In addition, the results of this research may encourage further study of non-specific effects for other types of ED interventions and for other types of conditions.

## Competing interests

All authors declare that they have no competing interests.

## Authors’ contributions

FF has the main responsibility of this review and will draft the protocol, develop the search strategy, search for studies, extract data into Review Manager and SPSS, carry out the analysis, interpret the analysis, and draft the final write-up of the review. FF and GEB will apply inclusion criteria and FF, GEB, and KS will extract data from studies. GEB, KS and DD will give feedback on subsequent protocol drafts and the publication manuscript. KS will assist with the preparation of the protocol manuscript. GEB and DD will assist where needed during the whole review process. All authors read and approved the final manuscript.

## Appendix 1

### PubMed/MEDLINE search strategy

1. 3^′^,5^′^ cyclic GMP phosphodiesterase (MeSH terms)

2. phosphodiesterase 5 inhibitors (MeSH major topic)

3. phosphodiesterase* (title/abstract)

4. phospho-diesterase* (title/abstract)

5. pde 5 (title/abstract)

6. sildenafil (text word)

7. viagra (text word)

8. vardenafil (text word)

9. levitra (text word)

10. tadalafil (text word)

11. cialis (text word)

12. 1 OR 2 OR 3 OR 4 OR 5 OR 6 OR 7 OR 8 OR 9 OR 10 OR 11

13. impotence (MeSH terms)

14. erecti* (title/abstract)

15. dysfunct* (title/abstract)

16. erect* (text word)

17. penile erection (MeSH terms)

18. sex* (title/abstract) AND 15

19. sex* (title/abstract) AND disorder* (title/abstract)

20. ((14 OR 16 OR 17) AND 15)

21. 13 OR 14 OR 16 OR 17 OR 18 OR 19 OR 20

22. randomized controlled trial (publication type)

23. controlled clinical trial (publication type)

24. randomized (title/abstract)

25. placebo (title/abstract)

26. drug therapy (MeSH subheading)

27. randomly (title/abstract)

28. trial (title/abstract)

29. groups (title/abstract)

30. 22 OR 23 OR 24 OR 25 OR 26 OR 27 OR 28 OR 29

31. animals (MeSH terms) not humans (MeSH terms)

32. 30 NOT 31

33. 2006/07/01 (date - publication): 3000 (date - publication)

34. 12 AND 21 AND 32 AND 33

## Appendix 2

### Screening questions for titles and abstracts

Randomized study?

Placebo control?

One PDE5 inhibitor type?

Primarily erectile dysfunction?

Sildenafil, vardenafil, or tadalafil?

Has IIEF outcome?

Is the language of the article English, German, French, or Dutch?

## Appendix 3

### Study eligibility form

Is this study a randomized controlled trial (RCT)?

Does this study investigate one type of PDE-5 inhibitor?

Does this study have a placebo comparison group?

Report primarily on treatment of erectile dysfunction?

Report on efficacy of either sildenafil, tadalafil, or vardenafil?

Report final assessment numerical data on IIEF-EF change scores OR (final AND baseline IIEF-EF scores) for placebo and intervention separately?

All men are diagnosed with ED?

All men are 18 years or older?

## Appendix 4

### Items to be included in the data abstraction forms

#### Identification study or report

Study ID

Report IDs

Review author ID

Study name

Country

### Study methods

Study design

Total duration of the double-blind phase of the study

Single-center vs. multi-center

Frequency and timing of follow-up for efficacy outcomes

Random sequence generation

Allocation sequence concealment

Blinding of patient

Blinding of caregiver

Blinding of outcome assessor

Blinding formally tested

Was ITT analysis performed?

Other concerns about bias

### Participants

Number of participants

Study setting

Comorbid condition

Age

Year of publication

Baseline ED severity IIEF-EF score

ED duration

Proportion of psychogenic ED etiology

PDE-5 inhibitor naive men

PDE-5 inhibitor responders

### Interventions

Control or intervention ratio

Run-in period: duration, intervention

For intervention group:

Type of PDE-5 inhibitor

Regimen (fixed or flexible dose)

Dose

For both intervention and control groups:

Compliance (number doses taken)

### Outcomes

For each outcome of interest:

Outcome definition

Unit of measurement

### Results

Baseline comparability of groups

Number of participants allocated to each intervention group

For each outcome:

Unit of analysis

Sample size

Missing participants

Summary data for each intervention group

Estimate of effect with confidence interval; standard deviation; *P* value; standard error

For AEs:

Sample size

Missing participants

AE type

Number of AEs

### Miscellaneous

Funding source

Assumptions made (outcome and specification)

Miscellaneous comments by review authors

## Abbreviations

AE: Adverse event; CI: Confidence interval; ED: Erectile dysfunction; EF: Erectile functioning; FDA: Food and Drug Administration; GEQ: Global efficiency question; IIEF: International Index of Erectile Functioning; ITT: Intention-to-treat; LOCF: Last observation carried forward; MCID: Minimal clinically important difference; MD: Mean difference; PDE-5: Phosphodiesterase-5; RCT: Randomized controlled trial; RR: Risk ratio\.
